# Redox Additive Electrolytes for Supercapacitors: A Mini-Review on Recent Developments and Future Directions

**DOI:** 10.3390/molecules30081764

**Published:** 2025-04-15

**Authors:** Lu Guan, Liangliang Guo, Haiyuan Yao, Jun Cai, Xuewei Dong, Ruonan Wang, Zhihua Zhai, Xuan Chen, Xiuzhi Wei, Dajin Li, Xingtong Liu, Shanshan Ji, Fanxiao Meng

**Affiliations:** 1 Department of Biological and Chemical Engineering, Jining Polytechnic, Jining 272037, China; gll@jnzyjsxy.edu.cn (L.G.); yhysan@jnzyjsxy.edu.cn (H.Y.); caijun_77@jnzyjsxy.edu.cn (J.C.); dxwsd@163.com (X.D.); wrn2024@163.com (R.W.); zhzhai_mail@163.com (Z.Z.); chen1996xuan@163.com (X.C.); 15092758899@163.com (X.W.); 15854637213@163.com (D.L.); 19819555290@163.com (X.L.); 2 College of Chemical Engineering, China University of Petroleum (East China), Qingdao 266580, China; 3 Faculty of Humanities, Altai Li University, Barnaul 656099, Russia; 4 State Key Laboratory of Applied Organic Chemistry, College of Chemistry and Chemical Engineering, Lanzhou University, Lanzhou 730000, China

**Keywords:** supercapacitors, redox additive electrolytes, aqueous electrolytes, non-aqueous electrolytes, solid-state electrolytes

## Abstract

Supercapacitors are promising energy storage devices that combine high power density, fast charge/discharge rates, and excellent cycling stability. However, their relatively low energy density compared to batteries remains a major challenge. To address this limitation, redox additive electrolytes have emerged as a key strategy to introduce reversible Faradaic reactions, significantly enhancing the energy storage capacity of supercapacitors. This mini-review systematically summarizes recent advancements in the use of redox-active species across aqueous, non-aqueous, and solid-state/gel electrolytes. We highlight the role of both inorganic and organic redox additives, detailing their mechanisms, advantages, and limitations in improving energy density and stability. Furthermore, we discuss the challenges associated with redox species, such as solubility, long-term stability, and parasitic side reactions, which hinder their practical applications. Future research directions are proposed to optimize redox-active materials and electrolyte systems, aiming to develop next-generation supercapacitors with superior energy density, extended cycling life, and enhanced applicability.

## 1. Introduction

**Figure 1:**
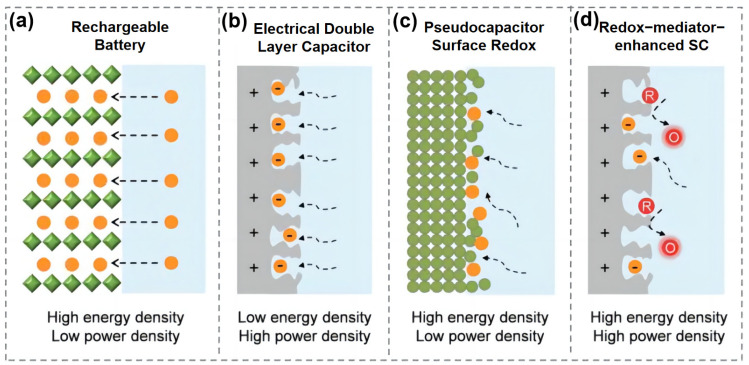
Schematic representation of charge storage mechanisms: (**a**) battery, (**b**) electric double-layer capacitor, (**c**) pseudocapacitor, and (**d**) redox-mediated supercapacitor [16].

Supercapacitors (SCs), also known as electrochemical capacitors, are emerging as essential energy storage devices due to their superior characteristics, such as high power density, rapid charge/discharge capability, and outstanding cycle life [1,2,3]. These properties make them particularly suitable for applications requiring instantaneous energy delivery, such as electric vehicles, renewable energy systems, and portable electronics [4,5]. Unlike conventional batteries, which store energy via bulk redox reactions (Figure 1a), SCs utilize electric double-layer capacitance (EDLC) and pseudocapacitance [6,7,8,9,10]. EDLC arises from electrostatic charge accumulation at the electrode–electrolyte interface (Figure 1b) [11,12,13], whereas pseudocapacitance originates from fast, reversible redox reactions at or near the electrode surface [14,15] (Figure 1c).

Despite their advantages, SCs suffer from low energy density compared to batteries, which hinders their widespread application in energy-intensive systems [17]. The energy density (E) of a supercapacitor is directly proportional to the square of the operating voltage (V) and the capacitance (C), as given by *E =* 1/2*CV*^2^. Thus, increasing the operating voltage and the overall charge storage capacity is critical to enhancing supercapacitor performance [18,19,20,21,22,23,24]. However, the electrochemical stability window (ESW) of the electrolyte fundamentally limits the operating voltage, presenting a significant bottleneck [25,26].

The electrolyte plays a decisive role in determining the electrochemical performance of SCs [27,28,29]. It provides ionic conductivity for charge transfer, defines the voltage window, and impacts the device’s energy density and stability. Electrolytes are broadly categorized into three types:

1. Aqueous electrolytes [30,31,32,33,34,35], such as H_2_SO_4_ and KOH, offer high ionic conductivity and cost-effectiveness but are restricted by a narrow voltage window (typically 1.0–1.2 V) due to water electrolysis.

2. Non-aqueous electrolytes [36,37,38,39,40,41], including organic solvents and ionic liquids (ILs), provide a broader voltage window (~3 V or more), enabling higher energy densities, but they suffer from lower ionic conductivity and higher cost.

3. Solid-state electrolytes [42,43,44,45,46,47], such as gel or polymer electrolytes, offer improved safety and flexibility but face challenges such as low ionic conductivity and poor performance at elevated temperatures.

**Figure 2:**
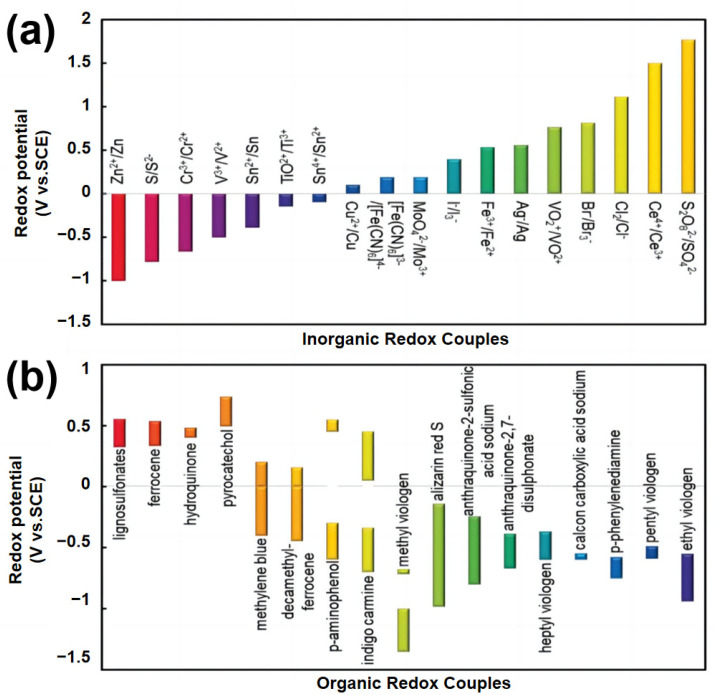
Redox potentials of various inorganic (**a**) and organic (**b**) redox electrolytes used for the construction of redox electrolyte-enhanced electrochemical capacitors [55].

Building upon the electrolyte challenges outlined above, the introduction of redox-active additives has emerged as a promising strategy to simultaneously address the dual constraints of low energy density and narrow voltage windows in supercapacitors [48,49]. By leveraging reversible oxidation–reduction reactions, these redox-active species create additional Faradaic charge storage pathways, effectively bridging the performance gap between traditional double-layer capacitors and batteries. As shown in Figure 2, redox-active electrolytes encompass both inorganic compounds (e.g., Fe^2+^/Fe^3+^, V^2+^/V^3+^) and organic molecules (e.g., quinones, viologens, 2,2,6,6-tetramethylpiperidinyloxyl derivatives), which exhibit varied redox potentials [50,51,52,53,54]. The appropriate selection of redox-active species is crucial, as their redox potential determines the contribution to the overall voltage window and energy output of the device. For instance, inorganic redox couples typically offer robust stability and fast redox kinetics, while organic molecules allow for tunable redox potentials and molecular design flexibility.

Recent research has demonstrated the effectiveness of redox additives across three major electrolyte systems:

- In aqueous electrolytes, redox-active species, such as K_3_Fe(CN)_6_ and NH_4_VO_3_, significantly expand the voltage window and improve energy density [56,57,58]. Their fast reaction kinetics and high solubility contribute to enhanced device performance.
- In non-aqueous electrolytes, organic redox mediators (RMs), like viologens and ferrocene derivatives, exploit the wide electrochemical stability of organic solvents and ILs, achieving higher voltages and energy densities [37,59].
- In solid-state electrolytes, redox-active gels or polymers incorporating species like hydroquinone and iodine-based mediators provide a balance between mechanical stability, safety, and improved charge storage [50,60,61,62].

Notably, research interest in redox-active electrolytes has increased dramatically in recent years. This highlights their potential as a transformative solution to the energy density limitations of conventional SCs. However, several challenges remain, including the stability of redox-active species over long-term cycling, their solubility in electrolytes, and the need to mitigate parasitic reactions that may occur during redox processes.

This mini-review aims to provide a concise yet comprehensive overview of recent advancements in the design, mechanisms, and performance of redox-active electrolytes across aqueous, non-aqueous, and solid-state systems. We systematically analyze the effects of redox species on supercapacitor energy and power densities, identify key challenges, and propose future research directions for the development of next-generation SCs.

## 2. Redox-Mediated Aqueous Electrolytes

Aqueous electrolytes, due to their high ionic conductivity, low cost, and environmental friendliness, are widely employed in SCs. However, their narrow ESW (typically 1.0–1.2 V) has significantly limited the achievable energy density [30,63]. Recent studies have demonstrated that incorporating redox-active additives into aqueous systems can introduce additional Faradaic charge storage, effectively expanding the operating voltage window and enhancing energy density. This section focuses on the advancements in inorganic and organic redox-active species, highlighting their mechanisms, benefits, and limitations in aqueous electrolyte-based SCs.

### 2.1. Inorganic Redox Additives in Aqueous Electrolytes

Inorganic redox additives have attracted considerable attention in recent years for their ability to enhance the performance of aqueous SCs. These additives, often based on transition metal compounds or other redox-active inorganic species, can expand the ESW, increase energy density, and improve cycling stability.

**Figure 3:**
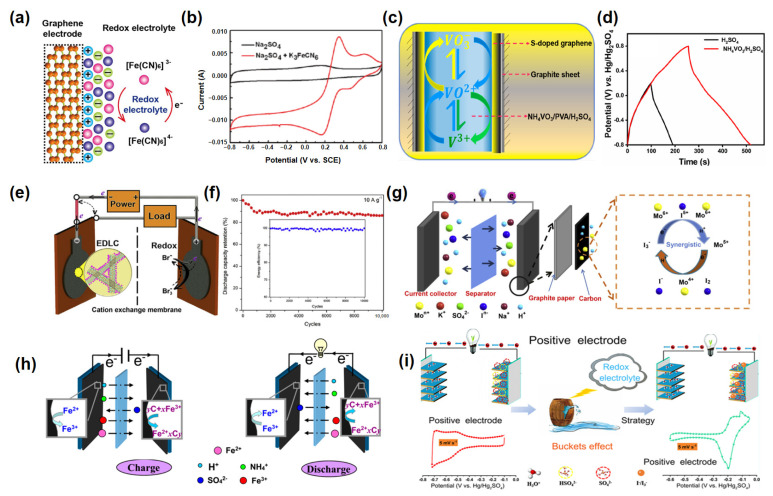
(**a**) Charge storage mechanisms of graphene paper electrode in the redox-electrolyte system and (**b**) cyclic voltammetry (CV) curves [64]. (**c**) Redox reaction mechanism of NH_4_VO_3_ as a redox additive in a supercapacitor. (**d**) Galvanostatic charge–discharge (GCD) of SSG-2 with H_2_SO_4_ and NH_4_VO_3_/H_2_SO_4_[65]. (**e**) The schematic illustration of the redox-active electrolyte supercapacitor. (**f**) Digital image of FCNT electrodes [66]. (**g**) The electrochemical reaction mechanism of the synergistic effects between Na_2_MoO_4_ and KI in the mixed electrolyte [67]. (**h**) Schematic drawing of the electrochemical reaction mechanism of FAS in the mixed electrolyte [68]. (**i**) Enhancement strategy for positive electrode in MXene-based symmetric SCs by redox electrolyte introduction [69].

Chen et al. first reported the use of potassium ferricyanide K_3_Fe(CN)_6_ as an inorganic redox additive [64]. The redox-active Fe(CN)_6_^3−^/Fe(CN)_6_^4−^ couple facilitated reversible Faradaic reactions (Figure 3a), which significantly increased the capacitance. A notable 5-fold improvement in specific capacitance was achieved compared to traditional EDLC systems alongside an expanded operating voltage window of 1.6 V, far exceeding the typical aqueous electrolyte limit (Figure 3b). This pioneering study underscored the feasibility of incorporating stable and robust redox couples into aqueous systems to boost energy storage capacity. Building on this concept, Sandhiya et al. investigated ammonium vanadate (NH_4_VO_3_) as a redox additive in H_2_SO_4_ electrolyte (Figure 3c) [65]. The addition of NH_4_VO_3_ led to a dramatic enhancement in specific capacitance (Figure 3d), increasing from 375 F g^−1^ to 658 F g^−1^, and improved the energy density to 32 W h kg^−1^—a 4.5-fold improvement over the baseline H_2_SO_4_ system. This enhancement was attributed to the redox activity of VO ions, which interact with H^+^ to deliver stable and reversible pseudocapacitance. The study underscored the importance of integrating transition metal-based RMs to overcome the inherent limitations of aqueous systems.

In an innovative approach, Tang et al. introduced a hybrid supercapacitor system using functionalized carbon nanotubes (FCNTs) and a neutral KBr-based redox-active electrolyte (Figure 3e) [66]. The incorporation of the Br⁻/Br_3_⁻ redox couple facilitated battery-like pseudocapacitive behavior on the positive electrode, while the FCNT electrodes retained electric double-layer behavior on the negative electrode. This hybrid system achieved a remarkable four-fold increase in energy density compared to traditional EDLCs using FCNTs in Na_2_SO_4_ electrolyte. A maximum energy density of 28.3 W h kg^−1^ was obtained at 0.5 A g^−1^ along with outstanding cyclic stability, maintaining 86.3% of its initial capacity after 10,000 cycles (Figure 3f). This work demonstrated the synergistic benefits of combining double-layer capacitance and redox-active pseudocapacitance in hybrid systems.

The synergistic effect of combining multiple redox species was further explored by Xu et al., who investigated a dual-additive system of sodium molybdate (Na_2_MoO_4_) and potassium iodide (KI) in H_2_SO_4_ (Figure 3g). The overlapping redox potentials of molybdate and iodide ions facilitated efficient charge transfer and contributed to a 17.4-fold increase in specific capacitance. The optimized system delivered an impressive energy density of 65.3 W h kg^−1^, demonstrating the potential of multi-species electrolytes to achieve higher energy storage capabilities [67].

Meanwhile, Sun et al. explored the use of ferrous ammonium sulfate (FAS) in H_2_SO_4_. The Fe^2+^/Fe^3+^ redox couple provided stable and reversible pseudocapacitance (Figure 3h), leading to an ultrahigh specific capacitance of 1499 F g^−1^ and an energy density of 58.7 W h kg^−1^. The study also emphasized the importance of optimizing the potential window to achieve superior performance, highlighting the critical role of electrolyte engineering in enhancing energy storage [68].

Tian et al. extended this concept by investigating MXene-based symmetric SCs with redox-active KI in H_2_SO_4_ (Figure 3i). The addition of KI transformed the positive electrode’s behavior from capacitive to battery-like, enhancing the energy storage capacity and resulting in a volumetric energy density of 33.2 W h L^−1^. This was a significant improvement over the 14.3 W h L^−1^ observed in pristine H_2_SO_4_ [69]. The study demonstrated the synergistic interaction of redox-active halides with advanced materials like MXenes, which are known for their high conductivity and excellent ion intercalation properties.

The integration of inorganic redox-active additives into aqueous electrolytes has demonstrated significant advancements in enhancing energy density, specific capacitance, and cycling stability. Redox species, such as K_3_Fe(CN)_6_, NH_4_VO_3_, Na_2_MoO_4_, KI, and FAS, have shown their ability to supplement traditional EDLC behavior with Faradaic charge storage mechanisms, enabling aqueous SCs to approach the performance of batteries. Hybrid systems, such as those incorporating Br^−^/Br^3−^and MXene-based electrodes, highlight the versatility of inorganic redox couples in diverse material platforms.

However, several challenges remain in the practical application of redox-active electrolytes. Long-term stability of redox species is a critical concern, as side reactions or chemical degradation can diminish performance over extended cycling. The solubility and optimal concentration of additives must be carefully controlled to avoid saturation or electrode passivation. Furthermore, the compatibility between redox-active species and electrode materials requires further optimization to maximize charge transfer efficiency and minimize parasitic reactions.

Despite these challenges, the strategic design and integration of inorganic redox additives provide a promising pathway for next-generation aqueous SCs. By addressing current limitations and leveraging advanced materials, future research can unlock the full potential of these systems, achieving higher energy density, improved stability, and broader applicability for energy storage technologies.

### 2.2. Organic Redox Additives in Aqueous Electrolytes

The introduction of organic redox-active additives into aqueous electrolytes has proven to be a powerful strategy for enhancing the energy density of SCs while preserving their inherent advantages of high power density, safety, and environmental compatibility. Compared to inorganic redox species, organic molecules offer substantial design flexibility, enabling tailored redox potentials, enhanced solubility, and improved interaction with electrode materials.

**Figure 4:**
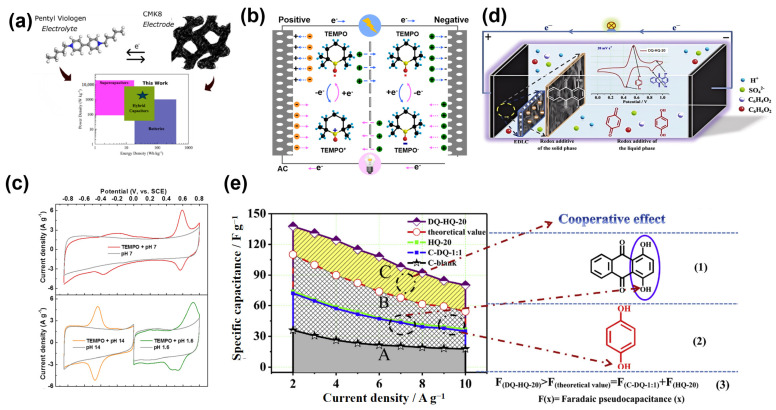
(**a**) Performance comparison of aqueous pentyl viologen/bromide redox-enhanced electrochemical capacitors with ordered mesoporous carbon (CMK-8) electrodes [70]. (**b**) Schematic diagram of the working mechanism of a supercapacitor with 2,2,6,6-tetramethylpiperidinyloxyl (TEMPO) as a bipolar redox mediator. (**c**) CV profiles of activated carbon (AC) electrodes in electrolyte with TEMPO under different conditions and schematic diagram of TEMPO redox reaction [71]. (**d**) Schematic diagram of the cooperative effect of 1,4-dihydroxyanthraquinone (DQ) and hydroquinone (HQ) in the carbon nanosheets-based supercapacitor. (**e**) Electrochemical mechanism of the cooperative effect of DQ and HQ in the DQ-HQ-20 sample [72].

The effectiveness of viologen-based redox systems has been exemplified in multiple studies. Calcagno et al. demonstrated the synergistic impact of combining pentyl viologen (PV) with bromide ions in aqueous electrolytes [70]. By employing CMK-8 electrodes, the system achieved a fine balance between diffusion-based redox reactions and surface adsorption (Figure 4a). The complementary redox activity of viologen and bromide ions extended the voltage window to 1.5 V, which significantly enhanced the system’s energy density while maintaining excellent cycling stability. This dual-phase system illustrated how combining two redox-active species can improve voltage utilization and maximize charge storage capacity.

Building on this, Wang et al. introduced an ambipolar organic radical, TEMPO, as a redox mediator capable of simultaneous oxidation and reduction at opposite electrodes (Figure 4b) [71]. Unlike conventional systems that rely on asymmetric redox activity, TEMPO exhibited symmetric Faradaic reactions, reducing energy losses and enhancing system efficiency. Figure 4c presents the CV profiles of AC electrodes with and without 1 mM TEMPO in different electrolytes at 10 mV s^−1^, illustrating the ambipolar characteristic of TEMPO and its relationship with the pH values of electrolytes. The electrolyte achieved an energy density of 51 W h kg^−1^, approximately 2.4 times higher than baseline systems without redox additives, while retaining 96% capacity after 4000 cycles. This ambipolar redox activity highlights the potential of TEMPO derivatives to simplify electrolyte design and optimize energy storage.

Complementary to viologens and radicals, quinone-based redox systems have been explored for their excellent redox kinetics and reversibility. Xu et al. developed a dual redox system integrating hydroquinone (HQ) in the electrolyte and 1,4-dihydroxyanthraquinone (DQ) within the electrode material (Figure 4d) [72]. Figure 4e shows that the specific capacitances of the A regions are the contribution of the electrochemical double-layer capacitor (EDLC), the B regions’ specific capacitances are derived from the theoretical sum of the pseudocapacitance of the C-DQ-1:1 and HQ-20 samples, and the pseudocapacitances of the DQ-HQ-20 sample are evidently higher than those of theoretical values with additional pseudocapacitances in the C regions. This work highlighted the advantage of combining solid-phase and solution-phase redox activity, where electrode-integrated redox species complement the solution-based mediator to maximize charge storage.

**Figure 5:**
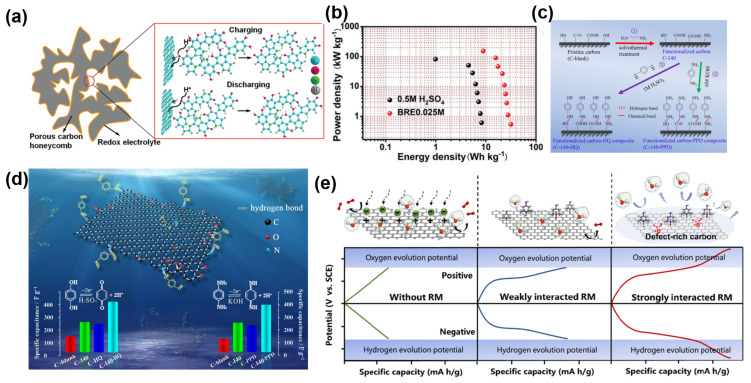
(**a**) Redox reaction involved with the BRE0.025 M electrolyte. (**b**) Comparison of energy densities in different systems [73]. (**c**) Schematic diagram of the interaction and reaction mechanism between ethylenediamine (EDA)-functionalized porous carbon and p-phenylenediamine (PPD)/hydroquinone (HQ). (**d**) Schematic diagram of the hydrogen bonding interaction between EDA-functionalized porous carbon and PPD/HQ [74]. (**e**) Schematic diagram of the effect of the interaction between RMs and defect-rich carbons on the voltage window and capacitance of aqueous SCs [75].

The concept of molecular engineering has enabled the design of advanced organic redox-active materials with improved charge transfer kinetics and long-term stability. Xiong et al. synthesized an indole-based conjugated macromolecule exhibiting a reduced HOMO-LUMO gap for faster electron transfer and enhanced stability [73]. Figure 5a presents the redox reaction involved with the BRE0.025M electrolyte, in which the 5,6-dihydroxyindole/5,6-quinoneindole endowed the BRE0.025M electrolyte with redox centers that involved the phenolic hydroxyl group in the 5,6-dihydroxyindole motifs and the quinonoid carbonyl group in the 5,6-quinoneindole motifs. When paired with honeycomb-like porous carbon electrodes, the system achieved exceptional performance, including a specific capacitance of 205 F g^−1^ at 1000 A g^−1^ and a power density of 153 kW kg^−1^ (Figure 5b), with capacitance retention of 97.1% after 20,000 cycles. This work underscores the importance of molecular design strategies in achieving high energy and power densities simultaneously.

The interaction between organic redox molecules and electrode surfaces can be enhanced through surface functionalization. As shown in Figure 5c, ethylenediamine was used to functionalize porous carbon, providing binding sites for redox additives. As shown in Figure 5d, the redox additive HQ was introduced and interacted with the functionalized carbon surface via hydrogen bonds. Zhai et al. demonstrated that amino-functionalized porous carbon electrodes improved the reactivity and stability of HQ in aqueous electrolytes. The functional groups on the electrode surface facilitated hydrogen bonding with HQ molecules, enhancing charge transfer and redox activity. This led to an energy density of 8.8 W h kg^−1^, a 3.2-fold increase over pristine carbon electrodes, highlighting the synergistic effects of optimized electrode–electrolyte interfaces in enhancing the performance of organic redox systems [74].

Guan et al. further advanced this concept by utilizing 4-hydroxy-TEMPO (4OH-TEMPO) with defect-rich carbon electrodes [75]. The strong interaction between 4OH-TEMPO and the electrode’s defect sites facilitated rapid electron transfer (Figure 5e), enabling a 67% increase in the voltage window and a six-fold improvement in specific capacitance. This study highlights the critical role of tailoring both RMs and electrode structures to achieve optimized energy storage performance.

In summary, organic redox additives, including viologens, ambipolar radicals, quinones, and conjugated macromolecules, have shown significant potential to address the energy density limitations of aqueous SCs. Systems that combine solution-phase and solid-phase redox species, such as hydroquinone–anthraquinone dual systems, or those that exploit symmetric Faradaic reactions, like TEMPO derivatives, illustrate the versatility of organic redox systems. Additionally, molecular engineering and surface functionalization strategies have demonstrated pathways to improve redox kinetics, stability, and charge transfer efficiency. Despite these advancements, challenges such as the chemical stability of organic redox species, solubility limitations, and parasitic side reactions under long-term operation remain unresolved. Future research efforts should focus on designing highly stable redox-active molecules, exploring synergistic redox combinations, and optimizing the electrode–electrolyte interface to fully realize the potential of organic redox additives in next-generation aqueous SCs.

The performance characteristics of several representative aqueous redox electrolyte systems are summarized in Table 1. These data provide a clear comparison of specific capacitance and energy density across different systems, further highlighting the significant potential of redox additives in enhancing the performance of aqueous supercapacitors. Additionally, Table 2 offers an overview and comparison of the advantages and disadvantages of inorganic and organic redox additives specifically used in aqueous electrolytes.

## 3. Redox-Mediated Non-Aqueous Electrolyte

Non-aqueous electrolytes [36,79], including organic solvent-based and IL systems, offer a significant advantage over aqueous counterparts due to their wide ESW, which can extend beyond 3 V [39,40]. This characteristic enables higher operational voltages, directly contributing to enhanced energy densities in SCs [80]. However, achieving high performance in non-aqueous systems requires overcoming challenges such as lower ionic conductivity, solvent volatility, and compatibility with redox-active species. This section explores recent advancements in redox-mediated non-aqueous electrolytes, with a focus on organic and IL-based systems, to address these limitations and unlock their potential for next-generation energy storage devices.

### 3.1. Redox-Mediated Non-Aqueous Organic Electrolytes

Redox-mediated non-aqueous organic electrolytes have emerged as a promising solution to enhance energy density in SCs, leveraging the broad voltage windows provided by organic solvents. By introducing redox-active organic mediators, such as viologens, quinones, and radical compounds, these systems can incorporate Faradaic charge storage alongside EDLC. Recent research has focused on the molecular design of redox species to optimize their stability, solubility, and charge transfer efficiency under high-voltage conditions.

**Figure 6:**
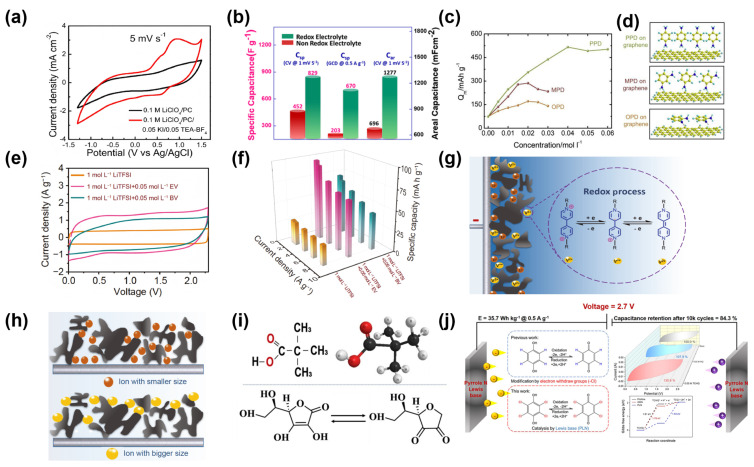
(**a**) CV comparison of Mn_0.95_Zn_0.05_Fe_2_O_4_ electrode in redox-active electrolyte (red) and non-redox-active electrolyte (black). (**b**) Specific capacitance performance of Mn_0.95_Zn_0.05_Fe_2_O_4_ in both electrolytes [81]. (**c**) The specific capacity of supercapacitor electrodes in 1 mol L^−1^ MeEt_3_NBF_4_ acetonitrile electrolyte with different concentrations of o-phenylenediamine (OPD), m-phenylenediamine (MPD), or p-phenylenediamine (PPD) at a current density of 1 A g^−1^. (**d**) Hypothetical illustrations of the interaction between various phenylenediamine molecules and the graphene surface [82]. (**e**) CV curves of the SCs with different electrolytes at a scan rate of 10 mV s^−1^. (**f**) The specific capacity values of SCs using different electrolytes under various current density conditions. (**g**) Illustration of the redox process of viologen compounds in SCs. (**h**) Depiction of the compatibility between electrode pores and ions [59]. (**i**) Chemical structure of PA and redox reaction of AA [83]. (**j**) Comparison of the electrochemical performance of SCs with different electrolytes at 2.7 V voltage and schematic diagram of the tetrachlorohydroquinone (TCHQ) redox reaction mechanism [84].

Arun Kumar et al. introduced transition-metal-substituted manganese ferrites (Mn_0.95_Zn_0.05_Fe_2_O_4_) as electrode materials in non-aqueous electrolytes (0.1 M lithium perchlorate/propylene carbonate) [81]. In their study, potassium iodide (KI) was utilized as a redox-active additive, which significantly improved the energy density by providing additional Faradaic charge storage. The system exhibited a high specific capacitance of 829 F g^−1^ (Figure 6a) and an energy density of 77.5 W h kg^−1^ in a symmetric pouch cell configuration (Figure 6b). This work demonstrated the potential of redox-active additives to boost the energy performance of non-aqueous electrolytes, but it also highlighted the importance of choosing compatible RMs that can effectively work with electrode materials. The successful integration of KI was crucial in expanding the ESW and improving overall device performance.

Building on these insights, Fang et al. examined the role of PPD as a redox-active mediator in organic electrolyte SCs [82]. PPD’s compatibility with graphene surfaces, aided by its para-positioned amino groups, resulted in enhanced pseudocapacitive behavior and better charge transfer efficiency. The addition of PPD at an optimal concentration of 0.04 mol L^−1^ increased the specific capacitance to 516 mA h g^−1^, significantly improving energy density (Figure 6c). The hypothetical image in Figure 6d indicates that the para-amino group of the PPD molecule introduces less steric hindrance during the interaction with the graphene surface and is more conducive to adsorption on the graphene surface than MPD and OPD, providing strong evidence for its role in improving the performance of the supercapacitor. This study not only emphasized the importance of molecular structure in designing redox-active additives but also illustrated how organic molecules could effectively be used to enhance the overall electrochemical performance of non-aqueous systems. By improving the interaction between the redox-active molecules and the electrode surface, this study set the stage for further development in the design of organic RMs.

This line of research was further advanced by Yue Niu et al., who investigated various viologen derivatives as redox-active additives in acetonitrile-based electrolytes [59]. They found that the molecular structure of viologens played a crucial role in determining the overall electrochemical performance. For instance, ethyl viologen (EV) exhibited superior performance compared to benzyl viologen (BV), which was attributed to EV’s smaller molecular size and better compatibility with the electrode pores (Figure 6e). The incorporation of EV led to an increase in specific capacitance to 73.0 mA h g^−1^ and improved energy density (Figure 6f), while BV, despite its smaller band gap, had lower performance due to its larger size and reduced ionic conductivity (Figure 6g,h). This study reinforced the idea that careful design of redox-active molecules, with a focus on molecular size and structure, is essential to optimizing non-aqueous electrolyte performance.

Another noteworthy contribution came from Ragib Shakil et al., who explored bio-based RMs, such as pivalic acid (PA) and ascorbic acid (AA), in non-aqueous electrolytes (Figure 6i) [83]. This study addressed the growing interest in sustainable energy storage solutions by demonstrating that AA could provide additional pseudocapacitance, enhancing the overall energy storage performance. The system achieved a specific capacitance of 308 F g^−1^ and an energy density of 15 W h kg^−1^, which, although lower than synthetic systems, highlighted the potential for bio-based redox additives in environmentally friendly energy storage technologies. The study also underscored the challenges of balancing electrochemical performance with sustainability, suggesting that bio-based additives could serve as an alternative to traditional synthetic RMs.

Wang et al. developed a high-voltage, high-energy-density supercapacitor by incorporating substituted hydroquinone (tetrachlorohydroquinone, TCHQ) as a redox mediator in organic electrolytes and using N-doped activated carbon (NAC 950) as the electrode material (Figure 6j) [84]. TCHQ, with its high redox potential, expands the electrochemical window to 2.7 V by avoiding the formation of quinhydrone-like by-products. The pyrrole nitrogen atoms in NAC 950 act as electrocatalytic centers, promoting the dehydrogenation of TCHQ and enhancing reaction reversibility. At 2.7 V, the device demonstrated a specific capacitance of 140 F g^−1^, an energy density of 35.7 W h kg^−1^, and stable cycling performance, retaining 84.3% of its capacitance after 10,000 cycles. This work provides a new strategy for designing high-voltage RMs and emphasizes the role of molecular design and electrode surface optimization.

Non-aqueous organic electrolytes have shown significant promise for improving supercapacitor energy density through redox-active additives, such as organic mediators and transition-metal complexes. These additives help to expand the ESW, thereby increasing the operational voltage and overall energy storage capacity. However, challenges persist in terms of stability, solubility, and compatibility with electrode materials. High-voltage RMs, such as TCHQ, show potential in further extending the voltage window, but issues like side reactions, slow kinetics, and long-term stability need to be addressed. Future research should focus on designing more stable redox-active species, optimizing the interface between electrolytes and electrodes, and improving the scalability and sustainability of these systems for practical supercapacitor applications.

### 3.2. Redox-Mediated Ionic Liquid Electrolytes

**Figure 7:**
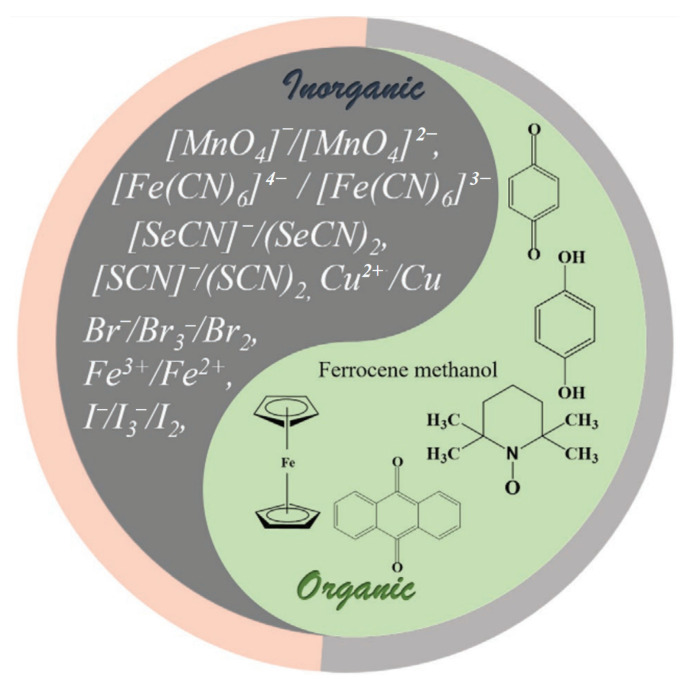
Summary of redox-active components in IL-REs [88].

ILs have attracted increasing attention as promising non-aqueous electrolytes for SCs due to their wide ESW, low volatility, and excellent thermal stability [85,86]. These intrinsic properties make ILs suitable for achieving high energy and power densities [87]. To further enhance their performance, redox-active species have been incorporated into ILs, forming redox-mediated ionic liquid electrolytes (RILEs). By introducing reversible Faradaic charge storage mechanisms, RILEs combine the advantages of ILs with the additional energy storage contribution from RMs, such as organic molecules and transition metal complexes. Despite challenges like low ionic conductivity and limited solubility of redox species, recent advancements have demonstrated the versatility and effectiveness of RILEs in enhancing charge storage capacity and expanding the operational voltage window. As summarized in Figure 7, these developments highlight innovative strategies for optimizing redox-active components, paving the way for next-generation high-energy SCs.

**Figure 8:**
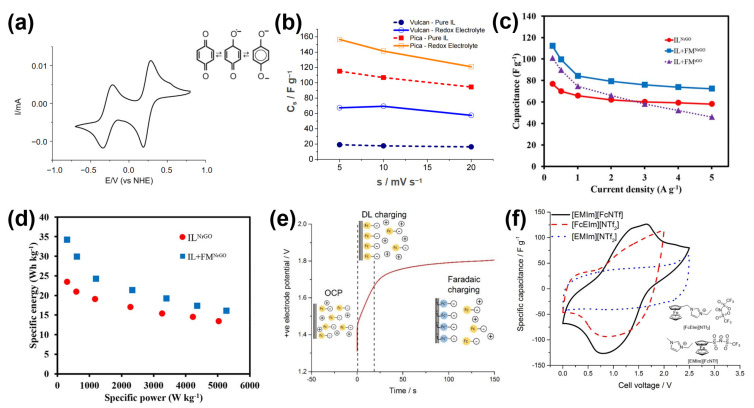
(**a**) CV of p-benzoquinone (p-BQ) in IL (PYR_14_TFSI). (**b**) Specific capacitance vs. scan rate curves of SCs with different electrodes and electrolytes [89]. (**c**) Calculated specific capacitance via GCDs of different combinations (NrGO with IL, NrGO with IL + FM, and rGO with IL + FM). (**d**) Ragone plots of NrGO with IL and IL + FM [90]. (**e**) The positive electrode’s charging potential profile exhibits the processes occurring during charging. (**f**) CV curves of two-electrode cells containing 80 wt.% IL in acetonitrile [91].

Recent research by Navalpotro et al. demonstrated the effectiveness of para-benzoquinone (p-BQ) as a redox-active additive in an IL electrolyte for hybrid SCs [89]. p-BQ introduced a two-step, one-electron reversible redox reaction, significantly enhancing electrochemical performance (Figure 8a). When Vulcan carbon was used as the electrode, the specific capacitance reached 70 F g^−1^ at 3 V with 0.4 M p-BQ, three times higher than the pure IL electrolyte (Figure 8b). This improvement is attributed to the additional charge storage from Faradaic reactions facilitated by p-BQ. However, when a high-surface-area carbon electrode (Pica carbon) was used, the capacitance increased by only 36%, suggesting that, in electrodes with larger micropores, the accessibility of redox species may be hindered by pore clogging at higher voltages. This highlights the importance of electrode material selection to maximize the benefits of redox-active additives in IL electrolytes.

Ma et al. explored the addition of ferrocene methanol as a redox mediator in a 1-butyl-1-methylpyrrolidinium dicyanamide IL electrolyte, using N-doped reduced graphene oxide aerogel (NrGO) electrodes [90]. Ferrocene methanol’s reversible redox reactions at the electrode–electrolyte interface enhanced both Faradaic charge transfer and the electric double-layer formation. The addition of 100 mM ferrocene methanol increased specific capacitance from 76.7 F g^−1^ to 112.1 F g^−1^ and specific energy from 23.5 W h kg^−1^ to 34.2 W h kg^−1^ (Figure 8c,d). The supercapacitor also showed good cycling stability, retaining 88.1% of its capacitance after 5000 cycles. This study demonstrates the potential of organometallic RMs to enhance the energy and power densities of IL-based SCs.

Expanding on redox-active ILs, Xie et al. synthesized two novel ionic liquids (RILs) by incorporating ferrocene into either the cation or anion [91]. These ferrocene units served as redox-active centers, contributing additional pseudocapacitance. As shown in Figure 8e, for the positive electrode, the charging process has a stage where the potential linearly rises as [FcNTf]⁻ anions fill the double layer (capacitive charging), and then a plateau occurs when the ferrocenyl unit on [FcNTf]⁻ oxidizes to [Fc⁺NTf]^0^, maintaining a stable potential up to 2 V. The supercapacitor using the RIL with ferrocene on the anion ([EMIm][FcNTf]) exhibited an energy density of 23.7 W h kg^−1^ at 2.5 V, an 83% increase compared to the unmodified IL (Figure 8f). The redox activity of the ferrocene-functionalized anion helped maintain a stable potential plateau during charging, suppressing self-discharge. In contrast, the RIL with ferrocene on the cation ([FcEIm][NTf_2_]) showed less improvement due to lower oxidation efficiency. This study emphasizes the role of molecular design in optimizing RILs for supercapacitor applications, suggesting that ferrocene-modified anions may provide better performance.

Previously, the effects of redox additives in non-aqueous organic electrolytes and ionic liquid electrolytes on the performance of supercapacitors have been introduced respectively. Different combinations of electrode materials, electrolytes, and redox additives can lead to varied effects. Table 3 summarizes the performance characteristics of some supercapacitors using non-aqueous redox electrolytes, which is helpful for a clearer comparative analysis of the performance differences among various systems.

## 4. Redox-Mediated Gel Electrolytes

Solid-state and gel polymer electrolytes [93,94,95,96] (GPEs) represent a promising solution for addressing the safety concerns of liquid electrolytes, offering improved mechanical stability, flexibility, and leakage resistance [97,98,99,100]. However, the energy density of these systems remains limited due to their reliance on EDLC alone. The integration of redox-active species into solid-state or gel electrolytes has emerged as a transformative strategy to introduce Faradaic charge storage, significantly improving energy density while maintaining system integrity.

**Figure 9:**
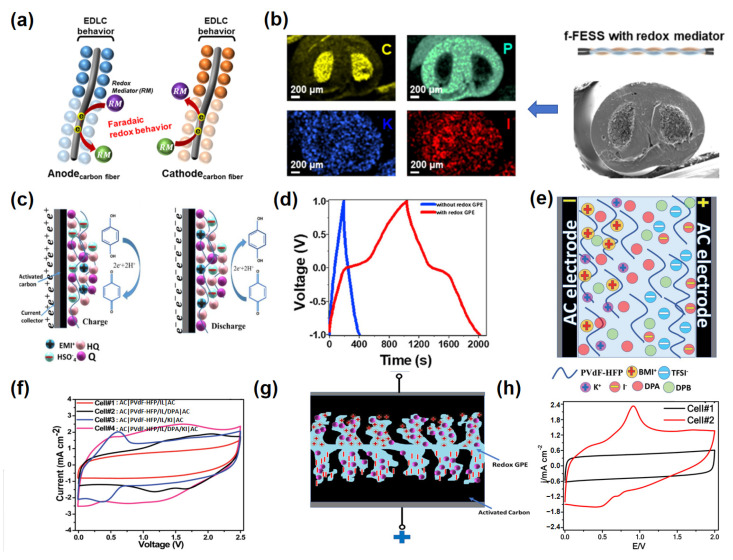
(**a**) Schematic representation depicting the Faradaic redox process mediated by the RM and the EDLC effect in the flexible fiber-shaped energy storage system (f-FESS) incorporating RM. (**b**) SEM and EDX mapping images of the f-FESS utilizing a redox-active KI polymer-gel electrolyte [101]. (**c**) Schematic diagram of the charge–discharge process of a supercapacitor with a redox-active electrolyte (containing hydroquinone). (**d**) Contribution of redox processes to the capacitance of the supercapacitor [102]. (**e**) Schematic illustration of the supercapacitor cell employing a redox-active gel polymer electrolyte (GPE) incorporating dual redox additives (PVDF-HFP/BMITFSI/DPA/KI) and AC electrodes. (**f**) CV curves of supercapacitor cells with different GPEs (Cell#1–Cell#4) at a 10 mV s⁻¹ scan rate for comparison [62]. (**g**) The SC cell schematic based on non-aqueous, redox-active GPE and AC electrodes. (**h**) Comparative CV curves of the capacitor cells (with and without redox-active GPE), recorded at a scan rate 5 mV s^−1^ [103].

Park et al. demonstrated a pioneering example of iodine-based RMs in a polymer-gel electrolyte [101]. By introducing potassium iodide (KI) into a flexible, solid-state electrolyte, the authors were able to achieve a three-fold enhancement in capacitance due to the synergistic contribution of KI’s Faradaic redox reactions and the double-layer capacitive behavior of carbon-based electrodes (Figure 9a). What made this work particularly notable was its emphasis on mechanical robustness, as the SC remained operational under extreme bending and twisting conditions (Figure 9b). This study set an important precedent for integrating redox-active species into flexible, wearable energy storage systems. However, it also revealed the challenges associated with iodine dissolution and potential self-discharge, both of which hinder long-term performance. The work highlights the need for stabilizing strategies to retain the redox mediator within the gel matrix.

Building on the concept of redox-active species, Yadav et al. explored the incorporation of organic mediators, specifically hydroquinone (HQ), into a quasi-solid-state GPE (Figure 9c) [102]. The choice of hydroquinone was driven by its well-known reversible redox activity, which provided a substantial boost in capacitance and energy density. By embedding HQ in a polyvinyl alcohol/polyvinylpyrrolidone (PVA/PVP) matrix, alongside the IL EMIHSO_4_, the authors not only achieved a high specific capacitance of 485 F g^−1^ but also demonstrated excellent electrochemical stability over 5000 cycles (Figure 9d). This work underscored the advantages of organic RMs, such as tunable redox potentials and strong Faradaic contributions, but it also brought to light critical limitations. Hydroquinone, while effective, suffers from solubility issues and gradual molecular degradation, which can compromise the long-term cycling stability of the device. The incorporation of ILs helped alleviate some of these issues by enhancing the ionic conductivity and electrochemical window, yet a more robust redox-active system remains necessary.

To address the inherent limitations of single RMs, Yadav and Hashmi extended their work to a dual redox system comprising diphenylamine (DPA) and potassium iodide (KI) [62]. This non-aqueous gel polymer electrolyte, based on PVDF-HFP and IL BMITFSI, demonstrated a synergistic effect between the two redox additives (Figure 9e). The complementary Faradaic reactions occurring at the positive and negative electrodes enabled the system to achieve an impressive energy density of 73.2 W h kg^−1^, far exceeding the capabilities of single-additive systems (Figure 9f). This work marked a significant advance by showing that dual-additive systems can overcome the capacity limitations of individual RMs while maintaining high power output. However, the complexity of such systems introduces challenges related to the precise optimization of redox potentials and mitigating potential cross-talk between reactions. The study also highlighted the need for improved understanding of redox mediator interactions within non-aqueous polymer matrices to ensure long-term stability and reproducibility.

Further advancing the field, Yadav et al. introduced a novel plastic-crystal-based GPE incorporating succinonitrile (SN) and hydroquinone in an IL (BMPTFSI) matrix (Figure 9g) [103]. This system not only exhibited excellent thermal stability and flexibility but also achieved a wide ESW, enabling a significant improvement in both capacitance (289 F g^−1^) and energy density (40 W h kg^−1^) (Figure 9h). The inclusion of succinonitrile, a plastic crystal known for its high ionic mobility and structural versatility, played a crucial role in enhancing ion transport while maintaining the mechanical integrity of the electrolyte. This work demonstrated the potential of hybrid systems that combine organic RMs with advanced electrolyte materials to achieve high-performance SCs. Nonetheless, challenges such as the cost of ILs and processability of plastic-crystal-based systems remain key barriers to large-scale commercialization.

Collectively, these studies illustrate the significant potential of redox-mediated GPEs in overcoming the energy density limitations of conventional SCs. In order to have a more comprehensive understanding of the application effects of gel redox electrolytes in supercapacitors, Table 4 summarizes some supercapacitors using such electrolytes and their performance characteristics. By introducing redox-active species into gel electrolytes, researchers have successfully combined pseudocapacitive Faradaic reactions with the physical charge storage of electric double-layer capacitors. In gel electrolytes, the gradual degradation and leakage of RMs present significant stability challenges, particularly for organic species. Additionally, the solubility and retention of mediators within the polymer matrix remain critical issues that need to be addressed to minimize self-discharge and capacity fade. Dual-additive systems offer a promising pathway to enhance performance, but their complexity introduces additional concerns regarding reaction optimization and cost. Future efforts should focus on the molecular engineering of RMs to improve stability as well as the development of cost-effective, scalable electrolyte systems. By addressing these challenges, redox-mediated gel electrolytes have the potential to enable next-generation SCs with superior energy density, enhanced cycling life, and broader applicability.

In the realm of supercapacitors, extensive research on electrolyte systems and redox additives has yielded numerous significant findings. Aqueous electrolytes are characterized by their low cost and high ionic conductivity, non-aqueous electrolytes are renowned for their wide voltage windows, and solid-state/gel electrolytes are recognized for their high safety and flexibility. Each of these electrolyte systems presents distinct advantages and application prospects (Table 5). As for redox additives, inorganic additives are typically associated with rapid electron transfer rates, while organic additives offer the advantage of adjustable redox potentials, thereby presenting diverse opportunities for enhancing supercapacitor performance (Table 6).

Notwithstanding these achievements, current research endeavors in this domain are confronted with several challenges. Aqueous electrolytes, despite their cost-effectiveness, are constrained by their narrow voltage windows, which inherently limit the enhancement of energy density. Non-aqueous electrolytes suffer from issues related to ionic conductivity and safety concerns. Additionally, the ionic conduction mechanisms of solid-state/gel electrolytes remain to be further elucidated and optimized. Redox additives, too, face formidable challenges, including stability issues, solubility limitations, and compatibility problems with electrode materials.

Looking towards the future, it is imperative to conduct in-depth investigations into the underlying mechanisms of electrolytes and additives. Leveraging advanced characterization techniques, such as in situ and operando spectroscopy and microscopy, can enable a precise analysis of the intricate relationship between their microstructures and properties. This, in turn, will provide a robust theoretical foundation for the rational design of novel materials. Simultaneously, the development of innovative composite systems holds great promise. For instance, integrating the unique advantages of different electrolyte systems and optimizing the synergistic interactions among additives, electrolytes, and electrode materials represent viable strategies to surmount the existing performance limitations. It is anticipated that, with continued research efforts, the performance of supercapacitors will witness substantial improvement, thereby meeting the ever-increasing demands for high-efficiency energy storage in emerging technologies, such as next-generation electronic devices, advanced electric vehicles, and grid-scale energy storage systems.

## 5. Conclusions

In summary, this review has highlighted the significant advancements in redox-active electrolytes for SCs, focusing on aqueous, non-aqueous, and solid-state systems. The incorporation of redox-active species across aqueous, non-aqueous, and solid-state systems has significantly advanced SC performance, particularly in achieving higher energy densities. In aqueous electrolytes, the use of inorganic redox additives, such as K_3_Fe(CN)_6_, NH_4_VO_3_, and KI, has demonstrated remarkable improvements in capacitance and energy density. Organic RMs, like viologens, TEMPO derivatives, and hydroquinones, offer additional flexibility in molecular design, enabling tailored redox potentials and synergistic charge storage behaviors. For non-aqueous electrolytes, high-voltage RMs, including phenylenediamine derivatives and hydroquinone substitutes, have extended the voltage window beyond 3 V, significantly increasing energy density while maintaining stability. Solid-state and gel polymer electrolytes incorporating redox-active species have further addressed safety concerns, offering flexible and robust energy storage solutions suitable for wearable and next-generation energy devices.

Despite these promising developments, critical challenges persist that hinder the practical application of redox-active electrolytes. Issues such as the limited long-term stability of redox species, parasitic side reactions, and solubility constraints remain unresolved. Furthermore, the compatibility between RMs and electrode materials requires further optimization to mitigate self-discharge and improve charge transfer efficiency. In non-aqueous and IL systems, the high cost and limited scalability of advanced electrolytes also pose significant barriers to commercialization.

To overcome these challenges, future research should focus on the rational design of highly stable redox-active molecules with enhanced solubility and minimized side reactions. The exploration of multi-redox additive systems and synergistic combinations of organic and inorganic species offers a promising pathway to further boost energy and power densities. Additionally, advances in interface engineering, molecular-level optimization, and the integration of emerging materials such as MXenes, conductive polymers, and nanostructured carbons will play a pivotal role in achieving high-performance, durable, and cost-effective SCs. Ultimately, redox-mediated electrolytes hold great promise as a transformative strategy to bridge the gap between SCs and batteries, paving the way for next-generation energy storage technologies with superior energy density, long cycling stability, and wide-ranging applicability.

## Figures and Tables

**Table 1:** Supercapacitors utilizing aqueous redox electrolyte and their performance characteristics.

Electrode Materials	Electrolytes	Redox Additives	Voltage of Cell (V)	Specific Capacitance	Energy Density	Ref.
Carbon fibers	KOH	KI	1.6	251 F g^−1^	7.1 W h kg^−1^	[76]
Carbon nanotube	Na_2_SO_4_	KBr	1.5	92.12 F g^−1^	28.3 W h kg^−1^	[66]
S doped graphene	H_2_SO_4_	HQ	1.6	300 F g^−1^	27 W h kg^−1^	[77]
Nanoporous carbon	Li_2_SO_4_	KI, Na_2_MoO_4_	1.0	470 F g^−1^	65.3 W h kg^−1^	[67]
Porous carbon	H_2_SO_4_	5,6-dihydroxyindole /5,6-quinoneindole	1.4	205 F g^−1^	8.8 W h kg^−1^	[73]
Waste charcoal carbon	KOH	PPD	1.0	512 F g^−1^	--	[78]

**Table 2:** Comparison of advantages and disadvantages of inorganic and organic redox additives in aqueous electrolytes.

Additive Type	Advantages	Disadvantages
Inorganic Redox Additives	1. High stability, maintaining stable performance over long periods. 2. Fast redox kinetics, enabling rapid charge and discharge. 3. Relatively low cost for some additives.	1. Fixed redox potential, with poor adjustability. 2. Moderate solubility, and high concentrations may cause saturation and precipitation, affecting performance. 3. Potential poor compatibility with electrode materials, leading to parasitic reactions and reduced capacitor efficiency.
Organic Redox Additives	1. Flexible molecular design, allowing for the adjustment of structures to achieve specific redox potentials. 2. High solubility, enabling better dispersion in aqueous electrolytes. 3. Good interaction with electrode materials, which can be further enhanced through surface functionalization.	1. Relatively poor stability, prone to chemical degradation during long-term cycling. 2. High cost for some organic additives. 3. Parasitic side reactions may occur, reducing energy efficiency and cycle life.

**Table 3:** Supercapacitors utilizing non-aqueous redox electrolyte and their performance characteristics.

Electrode Materials	Electrolytes	Redox Additives	Voltage of Cell (V)	Specific Capacitance	Energy Density	Ref.
Mn_0.95_Zn_0.05_Fe_2_O_4_	LiClO_4_/PC	KI	2.8	829 F g^−1^	77.5 W h kg^−1^	[81]
N-doped activated carbon	SBPBF_4_/PC	TCHQ	2.7	140 F g^−1^	35.7 W h kg^−1^	[84]
Reduced graphene oxide	TEABF_4_/ACN	PPD	3.0	340 F g^−1^	77.2 W h kg^−1^	[92]
Pica carbon	PYR_14_TSFSI	p-BQ	3.0	156 F g^−1^	30 W h kg^−1^	[89]
Activated carbon	LiTFSI/ACN	EV	2.3	73 mA h g^−1^	34 W h kg^−1^	[59]
Activated carbon	[FcEIm][NT]/ACN	Ferrocene	2.5	--	13.2 W h kg^−1^	[91]
N-doped reduced graphene oxide aerogel	[BMP][DCA]	Ferrocene methanol	3.0	112.1 F g^−1^	34.2 W h kg^−1^	[90]

**Table 4:** Supercapacitors utilizing gel redox electrolyte and their performance characteristics.

Electrode Materials	Electrolytes	Redox Additives	Voltage of Cell (V)	Capacitance	Energy Density	Ref.
Carbon fiber	H_3_PO_4_/KI/PVA	KI	1.0	461.8 F L^−1^	64.14 mW h L^−1^	[101]
Activated carbon	PVA/PVP/EMIHSO_4_	HQ	1.2	485 F g^−1^	24.7 W h kg^−1^	[102]
Activated carbon	PVDF-HFP/BMITFSI	NaI	1.5	334 F g^−1^	26.1 W h kg^−1^	[104]
Activated carbon	PVDF-HFP/SN/BMPTFSI	HQ	2.0	289 F g^−1^	40 W h kg^−1^	[103]
Carbon nanotubes	EMImTFSI/ADN	DmCc/DmCcPF_6_	3.1	57.1 F g^−1^	75.6 W h kg^−1^	[105]
Activated carbon	PVA/Li_2_SO_4_	BMIMI	1.5	384.1 F g^−1^	29.3 W h kg^−1^	[106]

**Table 5:** Comparison of electrolyte systems.

Electrolyte System	Characteristics	Ionic Conductivity	Voltage Window	Cost	Safety	Application Scenarios
Aqueous Electrolytes	High ionic conductivity; Environmentally friendly; Low cost	High	Narrow	Low	High	Large-scale energy storage, cost-sensitive applications
Non-aqueous Electrolytes	Wide voltage window; High energy density; High-voltage operation	Moderate	Wide	High	Medium	High-performance energy storage devices
Solid-state/gel Electrolytes	High safety; Good mechanical stability; Flexibility	Low	Wide	Medium	High	Wearable devices, flexible electronics

**Table 6:** Comparison of redox additive types.

Additive Type	Characteristics	Redox Potential	Stability	Solubility	Common Additives
Inorganic Additives	High stability; Fast redox kinetics	Fixed potential	High	Moderate	K_3_Fe(CN)_6_, NH_4_VO_3_, Na_2_MoO_4_, KI, FeSO_4_•(NH_4_)_2_SO_4_•6H_2_O, TiO_2_, etc.
Organic Additives	Flexible molecular design; High solubility	Tunable potential	Moderate	High	HQ, TEMPO, TCHQ, OPD, MPD, PPD, etc.

## Data Availability

No new data were created or analyzed in this study. Data sharing is not applicable to this article.
